# Prosocial Helping Behaviors, Genetic Risk for Alzheimer’s Disease, and Cognitive Decline in Later Life

**DOI:** 10.1093/geroni/igaf122.565

**Published:** 2025-12-31

**Authors:** Sae Hwang Han, Shiyang Zhang

**Affiliations:** The University of Texas at Austin, Austin, Texas, United States; The University of Texas at Austin, Austin, Texas, United States

## Abstract

Genetic factors play a critical role in the development of cognitive impairment and Alzheimer’s disease (AD), with individuals at high genetic risk—characterized by APOE ε4 status and broader polygenic risk—often experiencing symptom onset up to a decade earlier than those at low genetic risk. This early onset is partly driven by the accelerated cognitive decline associated with high genetic susceptibility to AD, yet little is known about protective lifestyle factors that may slow this progression. This study examines whether two forms of prosocial helping behaviors— volunteering through a formal organization and informal helping provided directly to friends and neighbors—attenuate the cognitive decline linked to genetic risk. The research questions were addressed using longitudinal data from individuals aged 51 and older in the nationally representative Health and Retirement Study (HRS; 1998–2020; N = 17,972; person-wave observations = 139,777). Cognitive function was assessed using a modified version of the Telephone Interview for Cognitive Status (TICS), and genetic risk was measured separately using APOE ε4 status and polygenic risk for AD, both available in the HRS. Preliminary findings from within-between random effects models indicate a robust within-person association between engagement in helping behaviors and a slower rate of cognitive decline, with particularly strong benefits observed among individuals at high genetic risk for AD. These results highlight the potential of prosocial engagement as a cost-effective public health intervention for delaying and preventing cognitive impairment and AD, particularly in high-risk populations.

